# Detrimental Actions of Chlorinated Nucleosides on the Function and Viability of Insulin-Producing Cells

**DOI:** 10.3390/ijms241914585

**Published:** 2023-09-26

**Authors:** Inga Sileikaite-Morvaközi, William H. Hansen, Michael J. Davies, Thomas Mandrup-Poulsen, Clare L. Hawkins

**Affiliations:** Department of Biomedical Sciences, University of Copenhagen, 2200 Copenhagen, Denmark; inga.sileikaite@sund.ku.dk (I.S.-M.); davies@sund.ku.dk (M.J.D.); tmpo@sund.ku.dk (T.M.-P.)

**Keywords:** chlorinated nucleosides, myeloperoxidase, hypochlorous acid, inflammation, diabetes

## Abstract

Neutrophils are innate immune cells that play a key role in pathogen clearance. They contribute to inflammatory diseases, including diabetes, by releasing pro-inflammatory cytokines, reactive oxygen species, and extracellular traps (NETs). NETs contain a DNA backbone and catalytically active myeloperoxidase (MPO), which produces hypochlorous acid (HOCl). Chlorination of the DNA nucleoside 8-chloro-deoxyguanosine has been reported as an early marker of inflammation in diabetes. In this study, we examined the reactivity of different chlorinated nucleosides, including 5-chloro-(deoxy)cytidine (5ClC, 5CldC), 8-chloro-(deoxy)adenosine (8ClA, 8CldA) and 8-chloro-(deoxy)guanosine (8ClG, 8CldG), with the INS-1E β-cell line. Exposure of INS-1E cells to 5CldC, 8CldA, 8ClA, and 8CldG decreased metabolic activity and intracellular ATP, and, together with 8ClG, induced apoptotic cell death. Exposure to 8ClA, but not the other nucleosides, resulted in sustained endoplasmic reticulum stress, activation of the unfolded protein response, and increased expression of thioredoxin-interacting protein (TXNIP) and heme oxygenase 1 (HO-1). Exposure of INS-1E cells to 5CldC also increased TXNIP and NAD(P)H dehydrogenase quinone 1 (NQO1) expression. In addition, a significant increase in the mRNA expression of NQO1 and GPx4 was seen in INS-1E cells exposed to 8ClG and 8CldA, respectively. However, a significant decrease in intracellular thiols was only observed in INS-1E cells exposed to 8ClG and 8CldG. Finally, a significant decrease in the insulin stimulation index was observed in experiments with all the chlorinated nucleosides, except for 8ClA and 8ClG. Together, these results suggest that increased formation of chlorinated nucleosides during inflammation in diabetes could influence β-cell function and may contribute to disease progression.

## 1. Introduction

Inflammation is a key innate immune defence characterised by the recruitment of immune cells, particularly macrophages and neutrophils, which are involved in the clearance of invading pathogens and repair of tissue [1]. However, chronic or prolonged unopposed inflammation is associated with the disruption of host cell functions and cellular damage and death, which underlies the development of numerous pathologies, including diabetes [1,2]. There is clinical proof-of-concept that pro-inflammatory cytokines, such as interleukin 1β (IL-1β) or tumour necrosis factor α (TNFα), released by macrophages, dendritic cells, and even β-cells themselves during pancreatic islet inflammation, contribute to the loss of pancreatic β-cell function in both type 1 diabetes (T1D) and type 2 diabetes (T2D) [2,3]. T2D is also characterised by systemic low-grade inflammation, which further contributes to β-cell decompensation by enhancing insulin resistance and the development of macrovascular and microvascular diabetic complications [3].

Neutrophils play a role in propagating inflammation and display dysregulated behaviour in diabetes [4]. The hyperglycaemic environment in diabetes promotes neutrophil degranulation and extracellular reactive oxygen species (ROS) formation, together with the release of proinflammatory cytokines and neutrophil extracellular traps (NETs) [4,5]. There is significant evidence linking aberrant NET release in T1D and T2D with disease complications, including compromised infection clearance, retinopathy, and development of cardiovascular disease [4,6]. In people with T2D, there are higher basal levels of NETs in the circulation compared to healthy controls [7,8], which remain elevated for up to a year following the normalisation of blood glucose concentration [9]. The increased susceptibility of neutrophils to undergo the release of NETs (termed NETosis) in T2D also impairs wound healing [5], which involves the increased activation of macrophages and a sustained inflammatory response [10]. However, the pathways by which NETs promote inflammation and host cell damage are complex and are not well understood [11].

NETs are web-like structures of DNA and histones, which contain the antimicrobial proteins myeloperoxidase (MPO) and elastase, and a range of other cellular proteins [12,13,14]. MPO catalyses the reaction of hydrogen peroxide with chloride ions to form the potent oxidant, hypochlorous acid (HOCl), which readily kills bacteria and other pathogens [15,16]. The DNA backbone of NETs provides a scaffold for MPO in the extracellular environment, which remains catalytically active and able to produce HOCl to detoxify pathogens that have evaded engulfment by phagocytosis [17]. However, HOCl also reacts rapidly with host cells and tissues and modifies most biological molecules, particularly proteins, DNA, and RNA, and is implicated in disease [18,19].

The reaction of HOCl with nucleic acids results in the formation of a range of chlorinated products, including short-lived *N*-chloramines (RR’N-Cl), and stable products containing a carbon–chlorine bond (C–Cl), such as 8-chloro-(2′-deoxy)-adenosine [8Cl(d)A], 8-chloro-(2′deoxy)-guanosine [8Cl(d)G], 5-chloro-(2′deoxy)-cytidine [5Cl(d)C], and 5-chloro-uracil [5ClUra] (reviewed [20]). There is evidence for the formation of stable chlorinated nucleosides under conditions of chronic inflammation in vivo (e.g., [21,22,23,24,25]), including in diabetes, where 8CldG is elevated in the urine [26]. The formation of chlorinated nucleosides has been attributed to HOCl-induced cellular damage [20,21], but it could also occur following the release of nucleic acids from necrotic cell death or NET release. Chlorinated nucleosides are mutagenic but also have a range of other biological effects, which are dependent on the nature of the nucleobase and the specific cell type [20]. In some cases, these reactions can promote the activation of stress-related signalling cascades and the propagation of inflammation [27,28,29]. There is also interest in their use as chemotherapeutic drugs, particularly 8ClA, which readily induces apoptosis in different malignant cell types [30,31,32,33].

Currently, it is not known whether chlorinated nucleosides can impair β-cell function or contribute to the development of diabetes. This may be important given the evidence for an elevation in NET release by neutrophils under diabetic conditions [4,5,7,8,9], and the close localisation of active MPO on the DNA backbone [12,13,14,17]. Moreover, in T1D, there is evidence for neutrophil infiltration into the pancreas, and increased NETosis, which occurs before the onset of clinical symptoms [34]. Therefore, in this study, we examined the hypothesis that chlorinated nucleosides would alter β-cell metabolism, function, and viability in a structure-dependent manner.

## 2. Results

### 2.1. Chlorinated Nucleosides Alter Metabolic Activity and Viability of INS-1E Cells in a Structure-Dependent Manner

**Figure 1:**
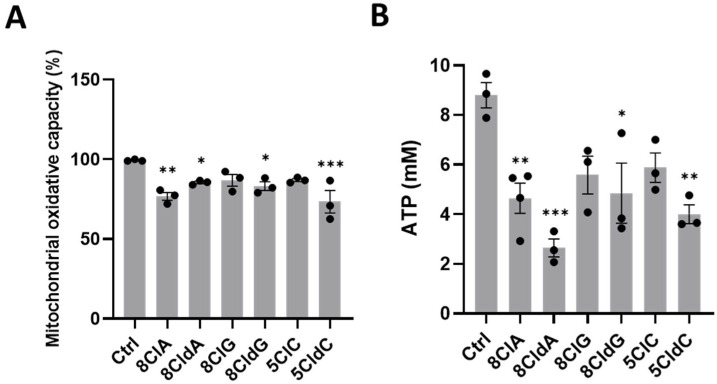
Effect of chlorinated nucleosides on metabolic activity and ATP levels in INS-1E cells. The INS-1E cells were exposed to 8ClA, 8CldA, 8ClG, 8CldG, 5ClC, and 5CldC (20 μM) for 24 h at 37 °C. Cell metabolic activity was assessed by the PrestoBlue assay (**A**). Intracellular ATP was measured using the ATPLite assay (**B**). Results represent the mean ± SEM of n = 3 experiments. The results are expressed relative to the untreated control. Significance compared to the untreated control was determined by one-way ANOVA with Dunnett’s post hoc test, * *p* < 0.05, ** *p* < 0.01, *** *p* < 0.001.

Initial experiments were performed to assess changes in cell metabolic activity after exposure of INS-1E cells to a range of chlorinated ribose and deoxyribose nucleosides, including 8ClA, 8CldA, 8ClG, 8CldG, 5ClC, and 5CldC (20 µM), for 24 h. A significant loss in cell metabolic activity was observed compared to the untreated control after exposure to 8ClA, 8CldA, 8CldG, and 5CldC but not 8ClG and 5ClC (Figure 1A). However, no changes to the metabolic activity of the cells were observed in the corresponding experiments with the non-chlorinated, parent nucleosides (Figure S1). Since the assay of metabolic activity probes mitochondrial oxidative capacity, these studies were extended to examine if the changes in metabolic activity correlated with a loss in ATP, particularly for 8ClA, which results in the formation of 8-Cl-ATP in a range of cell types [27,28,30,31,32,33]. Exposure of the cells to 8ClA, 8CldA, 8CldG, and 5CldC (20 µM) for 24 h resulted in a significant decrease in ATP, which was not observed in the corresponding experiments with 8ClG and 5ClC (Figure 1B). The ATP levels were not normalised to the protein concentration, so, in addition to reflecting the loss in metabolic activity, there could be some loss of ATP due to cell lysis.

**Figure 2:**
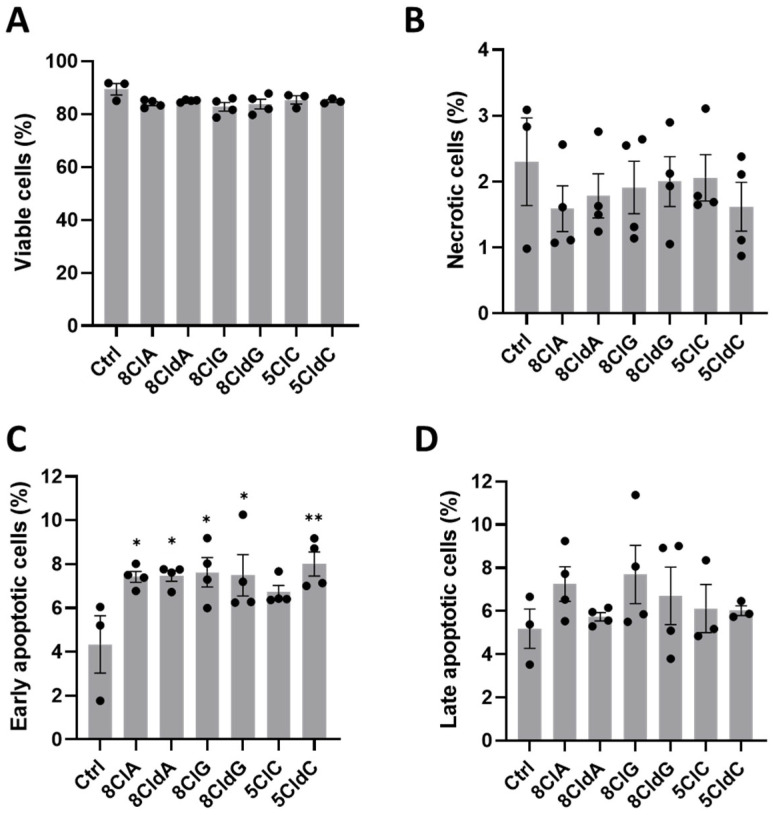
Exposure of INS-1E cells to chlorinated nucleosides induces cell apoptosis. The INS-1E cells were exposed to 8ClA, 8CldA, 8ClG, 8CldG, 5ClC, and 5CldC (20 μM) for 24 h at 37 °C before staining with propidium iodide (PI) and APC Annexin V and flow cytometry analysis. Graphs show the percentage of (**A**) viable cells (PI and Annexin V negative), (**B**) necrotic cells (PI positive, Annexin V negative), (**C**) apoptotic cells (PI negative, Annexin V positive), (**D**) late apoptotic cells (PI and Annexin V positive) in the total cell population. Results represent the mean ± SEM of n = 4 experiments. Significance compared to the untreated control was determined by one-way ANOVA with Dunnett’s post hoc test. * *p* < 0.05, ** *p* < 0.01.

To examine whether the loss of metabolic activity and ATP was indicative of decreased viability, flow cytometry was performed with Annexin V and propidium iodide (PI) staining for apoptotic and necrotic cell death, respectively. Exposure of the INS-1E cells to the chlorinated nucleosides (20 µM, 24 h) resulted in only small, non-significant changes in the live cell population (Figure 2A and Figure S2; Annexin V and PI negative). Similarly, there were no significant changes in the necrotic (Figure 2B and Figure S2; only PI positive) or late apoptotic (Figure 2D and Figure S2; Annexin V and PI positive) cell populations. However, a significant increase in the apoptotic (Annexin V positive) cell population was seen in experiments with 8ClA, 8CldA, 8ClG, 8CldG, and 5CldC (Figure 2C and Figure S2). In general, these data agree well with the results from the metabolic activity assay and changes in intracellular ATP. An exception was that a significant increase in the early apoptotic cell population was also seen with 8ClG, whereas the decrease in metabolic activity and ATP was not significant in this case.

### 2.2. Chlorinated Nucleosides Can Induce Stress-Related Signalling in INS-1E Cells

**Figure 3:**
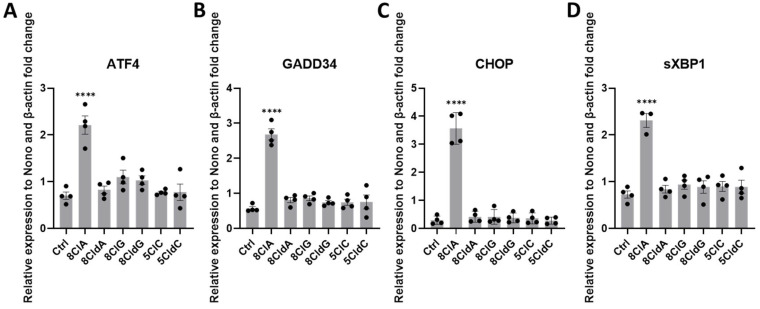
Exposure of INS-1E cells to 8ClA alters UPR gene expression in INS-1E cells. The INS-1E cells were exposed to 8ClA, 8CldA, 8ClG, 8CldG, 5ClC, and 5CldC (20 μM) for 24 h at 37 °C before the mRNA expression of (**A**) ATF4, (**B**) GADD34, (**C**) CHOP, and (**D**) sXBP1 were quantified using qPCR. Results are expressed as the fold change compared to the untreated control following normalisation to the average expression of housekeeping genes β-actin and Nono and represent the mean ± SEM of n = 4 experiments. Significance compared to the untreated control was determined by one-way ANOVA with Dunnett’s post hoc test. **** *p* < 0.0001.

Chlorinated nucleosides can activate different stress-related signalling cascades and alter antioxidant responses in different cell types [27,28,29], which could contribute to the observed changes in INS-1E cell function and viability. In particular, there is evidence that 8ClA can induce endoplasmic reticulum (ER) stress and alter the expression of different antioxidant and inflammatory genes [27,28]. Exposure of the INS-1E cells to 8ClA (20 µM) for 24 h resulted in a significant increase in the mRNA expression of activating transcription factor 4 (ATF4), growth arrest DNA damage-inducible protein 34 (GADD34), C/EBP homologous protein (CHOP), and spliced X-box binding protein 1 (sXBP1) (Figure 3). This is consistent with activation of the unfolded protein response (UPR) and induction of endoplasmic reticulum (ER) stress. There were no changes in the mRNA expression of these UPR-associated genes in the analogous experiments with 8CldA, 8ClG, 8CldG, 5ClC, or 5CldC (Figure 3). Similarly, there was no change in the expression of these UPR genes on exposure of the cells to non-chlorinated adenosine (Figure S3).

**Figure 4:**
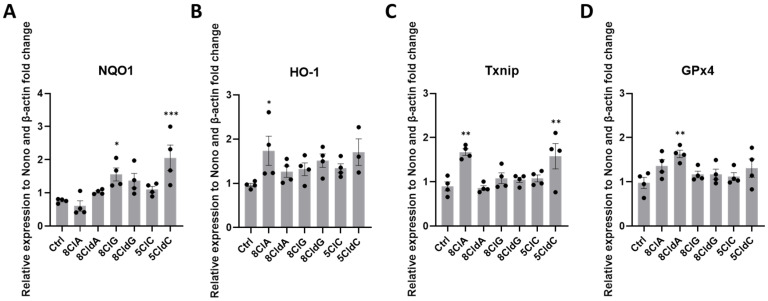
Effect of chlorinated nucleosides on antioxidant response gene expression in INS-1E cells. The INS-1E cells were exposed to 8ClA, 8CldA, 8ClG, 8CldG, 5ClC, and 5CldC (20 μM) for 24 h at 37 °C before the mRNA expression of (**A**) NQO1, (**B**) HO-1, (**C**) TXNIP, and (**D**) GPx4 were quantified using qPCR. Results are expressed as the fold change compared to the untreated control following normalisation to the average expression of housekeeping genes β-actin and Nono and represent the mean ± SEM of n = 4 experiments. Significance compared to the untreated control was determined by one-way ANOVA with Dunnett’s post hoc test, * *p* < 0.05, ** *p* < 0.01, *** *p* < 0.001.

Previous studies have indicated that ER stress can increase the expression of thioredoxin-interacting protein (TXNIP) [35] and induce an antioxidant response within cells through stimulation of the Nrf2 signalling pathway [36]. Therefore, we next examined the mRNA expression of TXNIP, together with heme oxygenase 1 (HO-1), NAD(P)H quinone oxidoreductase 1 (NQO1), and glutathione peroxidase 4 (GPx4), as examples of Nrf2-regulated genes [37]. Changes in mRNA expression were observed in the INS-1E cells exposed to 8ClA, and with 5CldC, 8ClG, and 8CldA (Figure 4). Exposure of the INS-1E cells to 8ClA resulted in a significant increase in the mRNA expression of TXNIP and HO-1, but not NQO1 or GPx4 (Figure 4). Again, no changes were observed with adenosine under analogous conditions (Figure S3). A significant increase in the mRNA expression of TXNIP was also observed in experiments with 5CldC (Figure 4C). However, with the 5CldC, there was an increase in NQO1 (Figure 4A), with the expression of HO-1 or GPx4 not significantly altered (Figure 4B–D). In addition, a significant increase in the mRNA expression of NQO1 and GPx4 was seen in INS-1E cells exposed to 8ClG and 8CldA, respectively (Figure 4).

**Figure 5:**
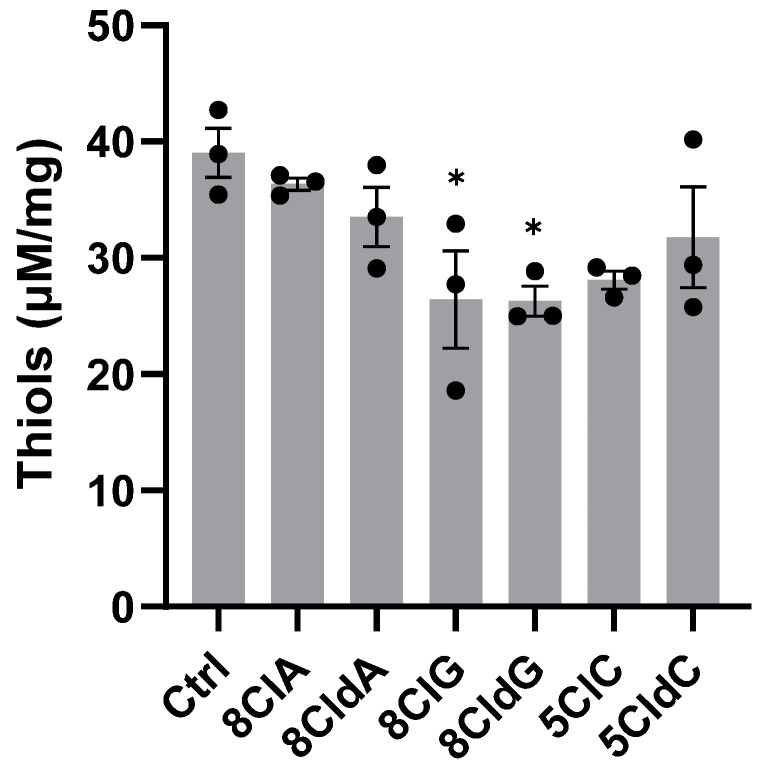
Effect of chlorinated nucleosides on thiol concentrations in INS-1E cells. The INS-1 cells were exposed to 8ClA, 8CldA, 8ClG, 8CldG, 5ClC, and 5CldC (20 μM) for 24 h at 37 °C. After the treatment, the cells were lysed with npH_2_O, and the thiol concentration in the lysates was determined using the ThioGlo1 assay. The results are shown as the thiol concentration normalised to the protein concentration and represent the mean ± SEM of n = 3 experiments. Significance compared to the untreated control was determined by one-way ANOVA with Dunnett’s post hoc test, * *p* < 0.05.

Given that there is a significant change in the expression of HO-1, NQO1, and TXNIP, which are sensitive to alteration in the cellular redox environment, the qPCR studies were extended to examine the effect of the chlorinated nucleosides on the concentration of intracellular thiols in the INS-1E cells, as a well-established marker of redox status in cells. The thiol concentrations were normalised to the protein concentration to correct for any loss in thiols as a result of cell lysis [38]. A significant decrease in intracellular thiol levels was only observed after exposure of the INS-1E cells to 8ClG or 8CldG, but not with 8Cl(d)A or 5Cl(d)C (Figure 5). Interestingly, there were no changes in the level of intracellular thiols on exposure of the cells to 8ClA or 5CldC, where a significant increase in the expression of UPR genes, HO-1, and TXNIP or NQO1, respectively, were seen.

### 2.3. Chlorinated Nucleosides Decrease Glucose-Stimulated Insulin Secretion by INS-1E Cells

**Figure 6:**
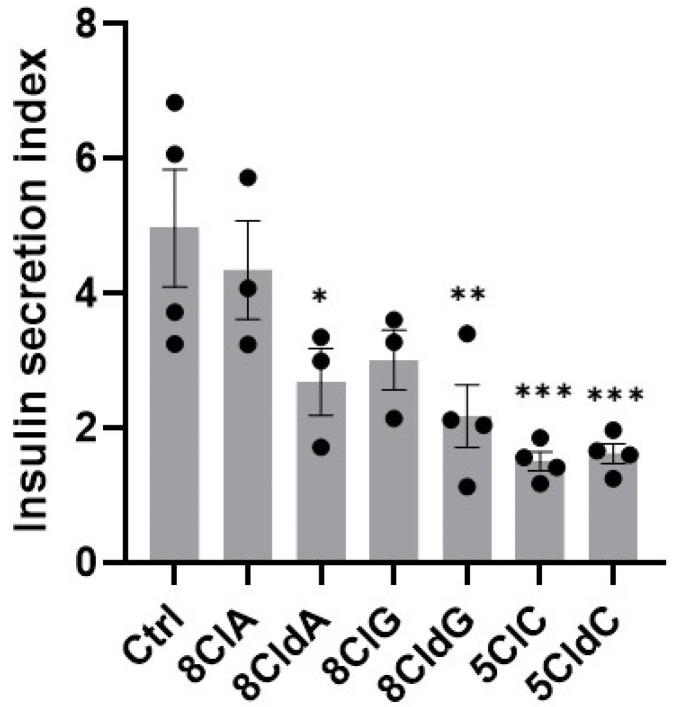
Effect of chlorinated nucleosides on insulin secretion in INS-1E cells. The INS-1 cells were exposed to 8ClA, 8CldA, 8ClG, 8CldG, 5ClC, and 5CldC (20 μM) for 24 h at 37 °C. The insulin secretion index was determined by measuring the amount of insulin secreted under high glucose stimulation (20 mM), divided by the basal insulin secreted in low glucose conditions (2 mM) by ELISA. Results are normalised to protein concentration and represent the mean ± SEM of n = 3–4 experiments. Significance compared to the untreated control was determined by one-way ANOVA with Dunnett’s post hoc test, * *p* < 0.05, ** *p* < 0.01, *** *p* < 0.001.

Experiments were also performed to examine whether the changes in metabolic activity, induction of apoptosis, and stress-related signalling influenced the secretion of insulin by the INS-1E cells. The INS-1E cells were exposed to each chlorinated nucleoside (20 µM) for 24 h before the glucose-stimulated insulin secretion was measured by ELISA. The insulin stimulation index, defined as the amount of insulin secreted under high glucose stimulation, divided by the basal insulin secreted in low glucose conditions, was then determined as a measure of β-cell functionality. The insulin stimulation index decreased significantly after exposure to 8CldA, 8CldG, 5ClC, and 5CldC, but not 8ClA or 8ClG (Figure 6). There was no change in the insulin content in the cell lysates on assessment by ELISA (Figure S4), or in the mRNA expression of the Ins1 or Ins2 genes on exposure of the INS-1E cells to any of the chlorinated nucleosides (Figure S4).

## 3. Discussion

There is evidence for the formation of chlorinated nucleosides under normal physiological conditions [24] and a range of pathological conditions (reviewed [20]). Chlorinated nucleosides have been used as biomarkers for systemic inflammation in different diseases, including diabetes, where urinary excretion of 8CldG is elevated [26]. Chlorinated nucleosides are formed by reaction with the MPO-derived oxidant, HOCl [20]. These products are observed on exposure of different cell types to HOCl [24,39,40], but may also be formed on the DNA backbone of NETs, given the presence of active MPO within these structures [17]. Neutrophils are more prone to release NETs under conditions of high glucose [5], the metabolic hallmark of diabetes and a key risk factor for its complications [6]. Given the elevation of NETs in the circulation of people with diabetes and the evidence of increased excretion of 8CldG, we examined whether different chlorinated nucleosides could alter β-cell function and metabolism. Using INS-1E cells as a β-cell model, we showed that exposure to chlorinated nucleosides resulted in alterations in metabolic activity, ATP levels, viability, stress-related signalling, and impairment of glucose-stimulated insulin secretion. These effects were dependent on the nucleoside structure, in accord with studies using other mammalian cell types (e.g., [27,29]).

The metabolic activity and ATP levels of INS-1E cells were decreased after exposure to 8ClA, 8CldA, 8CldG, and 5CldC for 24 h. These data contrast with studies with chlorinated nucleosides and other cell types, including macrophages [28] and human coronary artery endothelial cells (HCAEC) [27], where changes in metabolic activity and ATP levels were seen only with 8ClA. The reason for this is not certain but could reflect differences in the cellular uptake and/or incorporation of individual nucleosides into RNA or DNA, which was not measured here. However, it is also possible that differences in the metabolic profiles of each cell type could be a contributing factor. The changes in metabolic activity and ATP seen in β-cells exposed to 8ClA occurred concurrently with apoptotic cell death. It is well established that 8ClA is cytotoxic, particularly to malignant cells [31,32,41], which has led to its use as a chemotherapeutic drug [33,42], although it can also damage non-malignant cells [27,28]. 8ClA can be phosphorylated, resulting in 8Cl-ATP formation and a subsequent decrease in ATP, owing to competition of 8Cl-ADP with ADP and inhibition of mitochondrial ATP synthase [43]. 8ClA can also be succinylated, which depletes fumarate and reduces ATP production in malignant cells by inhibition of the citric acid cycle [42]. In addition, 8Cl-ATP inhibits the transcription and the activity of AMPK (5’ adenosine monophosphate-activated protein kinase) and mTORC1 (mammalian target of rapamycin complex 1), which decreases proliferation and influences nutrient sensing and energy homeostasis [44,45]. The formation of 8Cl-ATP was not measured here, but it is envisaged that a similar mechanism is responsible for the loss of ATP in INS-1E cells exposed to 8ClA, culminating in apoptotic cell death.

A loss of metabolic activity, decreased ATP levels, and apoptotic cell death were also observed with 8CldA, 8CldG, and 5CldC. Exposure to 8ClG increased apoptotic cell death, although, in this case, no significant changes in metabolic activity or ATP were observed. The mechanism responsible for toxicity in cells exposed to these chlorinated nucleosides is not known. With 8CldA, 8CldG, and 5CldC, the results could reflect the incorporation of the chlorinated nucleosides into cellular DNA, resulting in growth arrest, decreased proliferation, and cell death. The incorporation of chlorinated nucleosides into the DNA and RNA of the INS-1E was not examined here, but previous studies have shown that 5CldC readily incorporates into cellular DNA in other mammalian cell types [27,29]. This chlorinated nucleoside is also strongly mutagenic and implicated in the development of inflammatory cancers [46]. The incorporation of 8CldG and 8CldA into cellular DNA has been shown, but, in HCAEC, this occurred in the absence of toxicity at comparable nucleoside concentrations, perhaps as a result of repair mechanisms [27].

In addition to alterations in metabolism and DNA (or RNA) integrity, it is also possible that chlorinated nucleosides could affect cellular survival by the activation of stress-related signalling. With 8ClA, there was evidence for activation of the UPR and sustained ER stress, which may, together with 8Cl-ATP formation, contribute to the induction of apoptosis, as reported previously [27]. The increased expression of ATF4, GADD34, and the pro-apoptotic transcription factor, CHOP, are consistent with activation of the PERK branch of the UPR, although increases in sXBP mRNA are also seen, consistent with a contribution from the IRE1 signalling pathway [47]. Changes in UPR gene expression were not seen with the other chlorinated nucleosides, suggesting that other signalling pathways could also be involved in the perturbation of metabolism and cell death.

With 8ClA, the expression of HO-1 and TXNIP genes was also increased. The induction of HO-1 involves different redox-regulated transcription factors, including nuclear factor E2-related factor 2 (Nrf2), NFκB, and activator protein 1 (AP-1) [48]. In this case, there was no evidence for a change in NQO1 expression, which suggests that the induction of HO-1 may be independent of Nrf2 in INS-1E cells exposed to 8ClA, although PERK signalling and ER stress are reported to promote activation of Nrf2 via phosphorylation of the regulatory protein, Keap1 [36].

The induction of ER stress may also be involved in increasing the expression of TXNIP seen when treating INS-1E cells with 8ClA. Previous studies have reported an association between ER stress and TXNIP expression [35,49], including in cells exposed to 8ClA [27]. This is attributed to the ability of TXNIP to promote protein folding via regulating the activity of protein disulfide isomerases resident in the ER [50]. However, an increase in TXNIP expression was also observed in INS-1E cells exposed to 5CldC, where no induction of UPR signalling was observed. Again, this suggests that chlorinated nucleosides activate other stress-related signalling cascades, which needs further investigation. TXNIP is also associated with the induction of cell apoptosis via ER stress-independent pathways, including inflammasome assembly [51], and has been implicated in the driving β-cell apoptosis and complications in diabetes [52].

With 5CldC, an increase in NQO1 gene expression was observed, but this occurred in the absence of changes in HO-1. NQO1 is readily induced on exposure of cells to numerous cellular stressors, and, like HO-1, can be regulated both by Nrf2 and other redox-dependent pathways [53]. Similarly, the expression of NQO1 was significantly upregulated in INS-1E cells exposed to 8ClG in the absence of any changes in HO-1. It is well established that HO-1 and NQO1 play a key protective role against oxidative stress, including in diabetes and the prevention of diabetic complications [54,55]. Thus, overexpression of HO-1 reduces inflammation by decreasing the activation of pro-inflammatory transcription factors [54], whereas genetic knockdown of NQO1 is associated with insulin resistance in obese mice [55].

To examine whether the changes in gene expression occurred concurrently with alterations in redox homeostasis, intracellular thiol concentrations were measured in the INS-1E cells following exposure to each chlorinated nucleoside. A significant decrease in thiols was only noted with 8ClG and 8CldG. An increase in the mRNA expression of NQO1 was observed in experiments with 8ClG but not 8CldG. Moreover, there was no change in intracellular thiols in the INS-1E cells exposed to 8ClA, where HO-1 mRNA was elevated, or 5CldC, where NQO1 mRNA expression was increased. ThioGlo 1 allows the quantification of both protein-bound and low-molecular-mass thiols, including GSH. Therefore, it might be informative to specifically measure GSH and its oxidation product, GSSG, and also examine changes in thiol levels at earlier time points, which could precede the activation of stress-related signalling cascades.

Under inflammatory and hyperglycemic conditions, insulin secretion is impaired [56]. Glucose-stimulated insulin secretion was significantly decreased after exposure to 8CldA, 8CldG, 5ClC, and 5CldC for 24 h. In general, the decrease in insulin secretion corresponded with the decrease in metabolic activity and loss of ATP in the INS-1E cells. However, an exception was seen with 8ClA, where there was no change in the insulin stimulation index, despite evidence for activation of the UPR and apoptotic cell death. This contrasts with previous studies showing decreased insulin secretion on the induction of ER stress, and apoptosis in β-cells exposed to various pro-inflammatory cytokines [57]. This could reflect differences in the ratio of apoptotic cells to the total cell population, particularly as there was no significant difference in insulin content or the expression of Ins1 or Ins2 on the treatment of the INS-1E cells with any of the chlorinated nucleosides.

In summary, these results suggest that an elevation of chlorinated nucleosides in the circulation from neutrophil dysfunction under diabetic conditions could be detrimental to β-cell function and insulin secretion. The release of NETs from neutrophils is enhanced in diabetes [5], with NET degradation, as well as HOCl-induced cellular damage and nucleic acid release from necrosis, being a possible source of chlorinated nucleosides. Strategies to modulate NET release under inflammatory conditions could be a potential therapeutic approach, though this is challenging [58] and would not influence the release of chlorinated nucleosides from damaged cells. An alternative approach may therefore be to understand the specific stress-related signalling pathways responsible for altered gene expression, cell survival, and insulin secretion, which requires further study. Mitogen-activated protein kinase (MAPK) signalling could play a role in mediating these reactions, as seen when β-cells are exposed to pro-inflammatory cytokines, such as IL-1β [57]. Currently, there is limited information as to the concentration of chlorinated nucleosides present in vivo [25]. However, the concentrations used here are comparable to those formed in cells exposed to HOCl under physio-pathological conditions [39,40]. In addition, these results have implications for the clinical use of 8ClA as a chemotherapeutic drug, as the induction of ER stress and β-cell apoptosis was seen at concentrations comparable to those achieved in plasma following drug administration [59].

## 4. Materials and Methods

### 4.1. Reagents and Materials

All the aqueous reagents were prepared using nanopure water (npH_2_O) filtered through a four-stage Milli-Q system (Millipore, Burlington, MA, USA). All the reagents were from Sigma-Aldrich/Merck (Søborg, Denmark) unless stated otherwise. Chlorinated nucleosides (8ClA, 8CldA, 5ClC, 5CldC, 8ClG, and 8CldG) were obtained from BioLog Life Sciences Institute (Bremen, Germany) and reconstituted in npH_2_O to a stock concentration of 500 μM before storage at −80 °C. Chlorinated nucleosides for experimental exposures were prepared by diluting stock solutions into complete RPMI-1640 GlutaMAX medium to 20 µM and filtered through 0.22 μm syringe filters (VWR, Søborg, Denmark) to remove any contaminants prior to addition to the cells.

### 4.2. Cell Culture

The INS-1E insulinoma cells [60] were kindly supplied by Claes Wollheim (University Medical Center, Geneva, Switzerland). The cells were maintained in RPMI-1640 GlutaMAX culture medium (Thermo Fisher, Roskilde, Denmark) supplemented with 10% (*v*/*v*) fetal bovine serum (FBS; Thermo Fisher), 100 U mL^−1^ penicillin, 0.1 mg mL^−1^ streptomycin (Thermo Fisher), sodium pyruvate (1 mM; Thermo Fisher), HEPES buffer (10 mM; Thermo Fisher), and β-mercaptoethanol (50 μM). This culture medium is henceforth referred to as complete RPMI-1640. The INS-1E cells were cultured under sterile conditions in 75 or 175 cm^2^ tissue culture flasks in humidified 5% CO_2_ at 37 °C and were sub-cultured once per week. The experiments were performed with cells between passage numbers 60–78. Before use, all the reagents were warmed to 37 °C, unless stated otherwise.

### 4.3. Metabolic Activity and Cell Viability Studies

The metabolic activity of the INS-1E cells was examined using the PrestoBlue reagent. INS-1E cells (3 × 10^4^/well in a 96-well plate) were incubated with each chlorinated nucleoside (20 µM) in complete RPMI-1640 for 24 h at 37 °C, 5% CO_2_. Following exposure, the cells were washed with 37 °C HBSS before the addition of complete RPMI-1640 (100 µL) and 10 µL of PrestoBlue reagent (Thermo Fisher) and incubation at 37 °C for 1 h. Fluorescence was measured with λ_ex_ 560 nm and λ_em_ 590 nm using a SpectraMax i3 plate reader (Molecular Devices, Wokingham, UK). The pathway of cell death was examined using flow cytometry with propidium iodide (PI) and allophycocyanin (APC)-conjugated Annexin V (BioLegend, Nordic Biosite, Copenhagen, Denmark). The INS-1E cells (4.8 × 10^5^/well in a 6-well plate) were exposed to each chlorinated nucleoside (20 µM) in complete RPMI-1640 and incubated for 24 h at 37 °C, 5% CO_2_. After exposure, the cells were harvested using trypsin/EDTA solution (Thermo Fisher) and resuspended in a binding buffer (100 µL) containing APC Annexin V (5 µL) and PI (10 µL), as per the manufacturer’s instructions. The samples were incubated in the dark at 21 °C for 15 min and analysed with a FACS Calibur flow cytometer (BD Biosciences, Lyngby, Denmark), with 10,000 cells recorded for each sample. Data were analysed using FlowLogic 7.3 software.

### 4.4. Quantification of ATP

ATP was quantified by luminescence using the ATPlite assay system (Perkin Elmer, Ballerup, Denmark). The INS-1E cells (1.2 × 10^5^/well in a 24-well plate) were exposed to each chlorinated nucleoside (20 µM) in complete RPMI-1640 and incubated for 24 h at 37 °C, 5% CO_2_. Following exposure, the cells were washed, detached by gentle scraping, and resuspended in HBSS at a final volume of 250 µL. Each sample (100 µL) was transferred to a black 96-well plate, followed by the addition of the supplied cell lysis solution (50 μL) and mixing on an orbital shaker for 5 min. Luminescence was measured using a SpectraMax i3 plate reader (Molecular Devices) after the addition of the substrate solution (50 μL), mixing on the orbital shaker for 5 min, and dark adaption for 10 min.

### 4.5. Quantitative Real-Time Polymerase Chain Reaction (qPCR)

INS-1E cells (4.8 × 10^5^/well in a 6-well plate) were exposed to each chlorinated nucleoside (20 µM) in complete RPMI-1640 and incubated for 24 h at 37 °C, 5% CO_2_. RNA was extracted using an RNAeasy Mini Kit (Qiagen, Hilden, Germany) according to the manufacturer’s instructions, including a DNase digestion step (RNase-Free DNase Set, Qiagen). cDNA synthesis was achieved using a SensiFAST cDNA Synthesis Kit (Bioline, London, UK) according to the manufacturer’s instructions. Real-time qPCR was performed using a SensiFAST SYBR Hi-ROX kit (Bioline) on a QuantStudio 5 Real-Time PCR System (Thermo Fisher) under the following thermal cycling conditions: 95 °C for 10 min, then 95 °C for 15 s, 60 °C for 1 min for 45 cycles, followed by 95 °C for 15 s, 60 °C for 15 s, and 95 °C for 15 s. A melt curve step consisting of step-wise temperature increases of 0.5 °C every 5 s beginning at 65 °C and ending at 95 °C was performed. The primer sequences are shown in Table S1. Relative mRNA concentrations of the genes of interest were normalised to Non-POU domain-containing octamer-binding protein (Nono) and β-actin housekeeping genes. Data analysis was carried out using the 2^−ΔΔCT^ method.

### 4.6. Quantification of Cell Thiols

INS-1E cells (1.2 × 10^5^/well in a 24-well plate) were exposed to each chlorinated nucleoside (20 µM) in complete RPMI-1640 and incubated for 24 h at 37 °C, 5% CO_2_. After the exposure, the concentration of intracellular thiols was determined using the ThioGlo 1 reagent (Berry & Associates, Dexter, MI, USA) as described previously [38]. The concentration of thiols was determined using a standard curve constructed with GSH standards and normalised to the protein concentration determined using the Pierce BCA protein assay (Thermo Fisher). The fluorescence of ThioGlo 1 was measured using λ_ex_ 384 nm and λ_em_ 513 nm using a SpectraMax i3 plate reader (Molecular Devices).

### 4.7. Quantification of Insulin Secretion

Insulin secretion was assessed using a Rat Insulin ELISA kit (Mercodia, Uppsala, Sweden). INS-1E cells (1.2 × 10^5^/well in a 24-well plate) were exposed to individual chlorinated nucleosides (20 µM) in complete RPMI-1640 and incubated for 24 h at 37 °C, 5% CO_2_. Glucose-stimulated insulin secretion was examined by incubating cells with Krebs-Ringer buffer supplemented with 2 mM glucose for 30 min followed by a 30 min exposure to 20 mM glucose. The supernatants were collected and analysed according to the manufacturer’s protocol. Briefly, 10 µL of samples and standards were added to the coated 96-well plate. One hundred µL of enzyme conjugate was added to each well, and the plate was incubated on a plate shaker at 800 rpm for 2 h. After incubation, the plate was washed with the supplied washing buffer before the addition of the TMB substrate solution (200 µL/well) to each well and 15 min incubation at 21 °C. The absorbance was recorded on a SpectraMax i3 plate reader (Molecular Devices) at 450 nm after the addition of the Stop solution (50 µL/well).

### 4.8. Statistical Analysis

Statistical analyses were performed using GraphPad Prism software (v9.3; GraphPad Software, San Diego, CA, USA) using one-way ANOVA with Dunnett’s multiple comparison post hoc test, with *p* < 0.05 taken as significant.

## Data Availability

Data will be made available on request.

## Supplementary Materials

The following supporting information can be downloaded at: https://www.mdpi.com/article/10.3390/ijms241914585/s1.
