# Supplementary material for: Detrimental Actions of Chlorinated Nucleosides on the Function and Viability of Insulin-Producing Cells
Source: Int J Mol Sci. 2023 Sep 26;24(19):14585. doi: 10.3390/ijms241914585 (PMC10572493; doi:10.3390/ijms241914585)
Supplement: Supplementary file 1 [file ijms-24-14585-s001.zip › ijms-2552588-supplementary.pdf]

SUPPLEMENTARY MATERIALS

Detrimental Actions of Chlorinated Nucleosides on Function and Viability of Insulin-Producing Cells.

Inga Sileikaite-Morvaközi, William H. Hansen, Michael J. Davies, Thomas Mandrup-Poulsen and Clare L. Hawkins*

Department of Biomedical Sciences, University of Copenhagen, Panum, Blegdamsvej 3B, Copenhagen N, DK-2200, Denmark.

---

*Corresponding author: Clare Hawkins email: clare.hawkins@sund.ku.dk, Department of Biomedical Sciences, University of Copenhagen, Panum, Blegdamsvej 3B, Copenhagen N, DK-2200, Denmark

Supplementary Table S1. Housekeeping and target gene primer sequences.

**Gene**	Forward sequence (5'-3')	Reverse sequence (3'-5')
**NONO**	CCTGATGCGAGAGAACAAGAGA	CTGGACGGTTGAATGCAGGA
**β-actin**	ATCAAGATCATTGCTCCTCCTG	CAGCTCAGTAACAGTCCGCC
**ATF4**	GTTGGTCAGTGCCTCAGACA	CATTCGAAACAGAGCATCGA
**GADD34**	CCTTGATGTGGAAGCCCAAAGTT	TCCACTTCTTGCTCTCTAAGGCCAT
**CHOP**	CAGCGACAGAGCCAAAATAAC	TGTGGTGGTGTATGAAGATGC
**sXBP1**	CTGAGTCCGAATCAGGTGCAG	ATCCATGGGAAGATGTTCTGG
**HO-1**	CGACAGCATGTCCCAGGATT	TCACCAGCTTAAAGCCTTCCC
**SOD1**	GGTGCAGGGCGTCATTCACT	GAGTCTGAGACTCAGACCAC
**SOD2**	TGTAGAGCATTGCAGCACTG	CAGTAGAACAGGATTACAGC
**GPx4**	CCGGCTACAATGTCAGGTTT	ACGCAGCCGTTCTTATCAAT
**NQO1**	CAGAAACGACATCACAGGGGA	AGCACTCTCTCAAACCAGCC
**TXNIP**	AGTTACCCGAGTCAAAGCCG	GTTCTCACCTGTAGGCTGGTC
**INS 1**	GGGGAACGTGGTTTCTTCTAC	CCAGTTGGTAGAGGGAGCAG
**INS 2**	CAGCACCTTTGTGGTTCTCA	CACCTCCAGTGCCAAGGT

Supplementary Figure S1

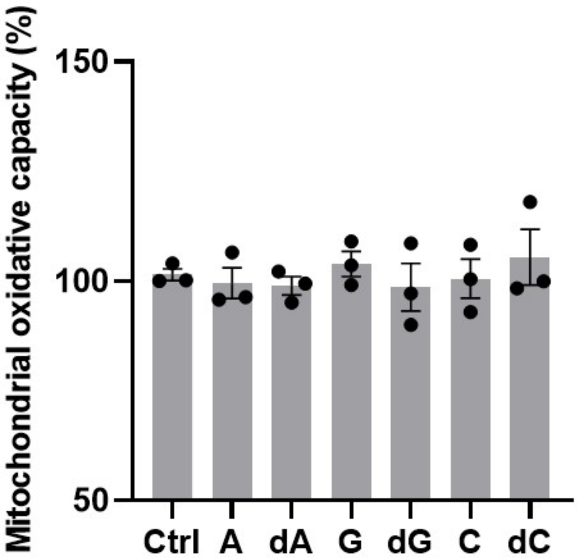

Figure S1. Non-chlorinated nucleosides do not affect the metabolic activity of INS-1E cells. The INS-1 cells were exposed to adenosine (A), 2-deoxyadenosine (dA), guanosine (G), 2-deoxyguanosine (dG), cytidine (C), and 2-deoxycytidine (dC) (20 µM) for 24 h at 37 °C. Cell metabolic activity was assessed by the PrestoBlue assay. Results represent the mean ± SEM of n = 3 experiments. The results are expressed relative to the untreated control. There were no significant differences compared to untreated controls by one-way ANOVA with Dunnett's post hoc test.

Supplementary Figure S2

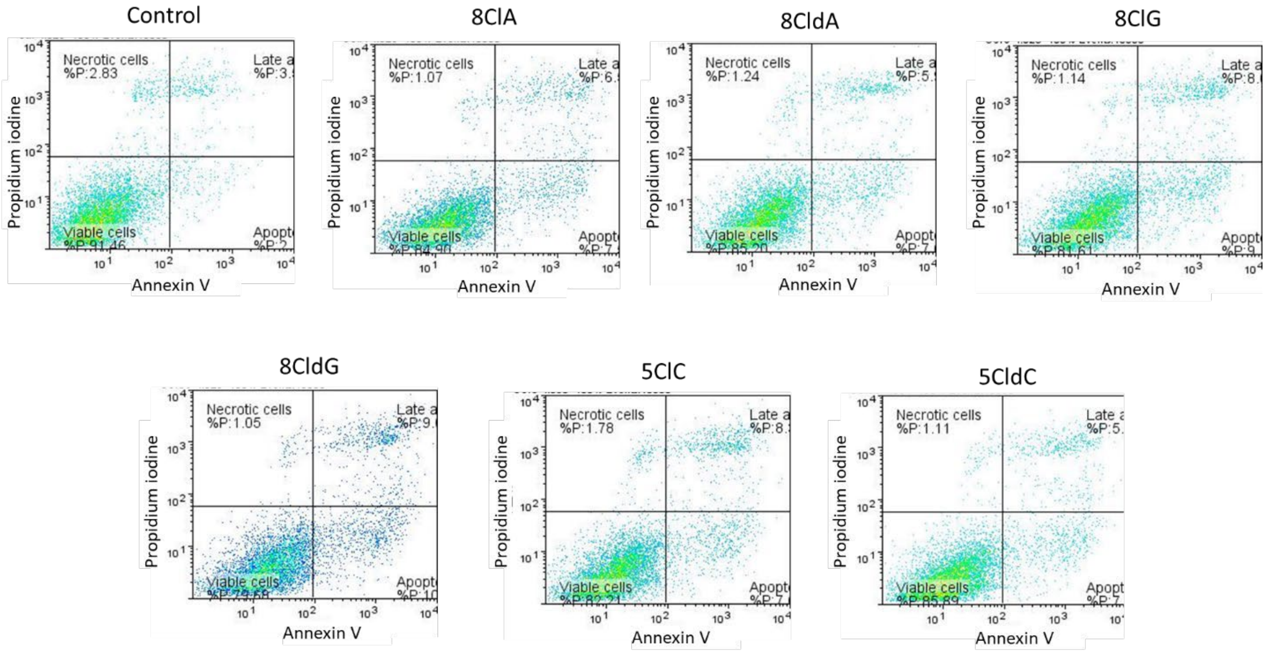

Figure S2. Exposure of INS-1E cells to chlorinated nucleosides induces cell apoptosis. The INS-1E cells (4.8x10^5 cells/well) were exposed to 8ClA, 8CldA, 8ClG, 8CldG, 5ClC, and 5CldC (20 µM) for 24 h at 37 °C. Data show representative flow cytometry dot plots after staining with Annexin V-APC and propidium iodide (PI). In each plot, the lower left quadrant shows live cells (negative for Annexin V and PI), upper left quadrant shows necrotic cells (positive for PI), lower right quadrant shows early apoptotic cells (positive for Annexin V, negative for PI), upper right quadrant shows late apoptotic (positive for Annexin V and PI).

Supplementary Figure S3

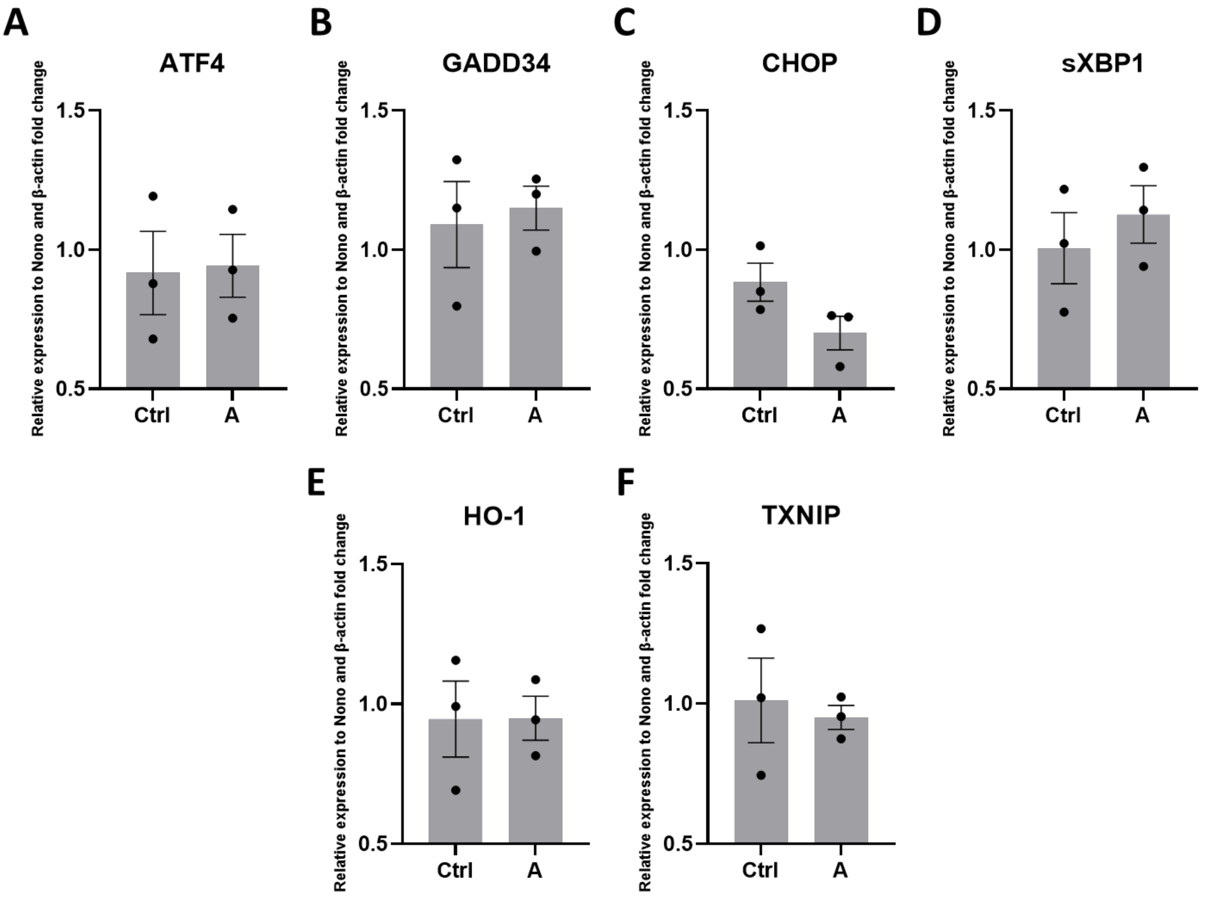

Figure S3. Adenosine does not affect UPR gene and antioxidant gene response in INS-1E cells. The INS-1 cells were exposed to adenosine (labelled "A", 20 µM) for 24 h at 37 °C. The expression of ATF4 (A), GADD34 (B), CHOP (C), sXBP1 (D), HO-1 (E), and TXNIP (F) genes was assessed using qPCR. Results are expressed as the fold change compared to untreated control following normalization to housekeeping genes β-actin and Nono and represent the mean ± SEM of n = 3 experiments. There were no significant differences compared to untreated controls by one-way ANOVA with Dunnett's post hoc test.

Supplementary Figure S4

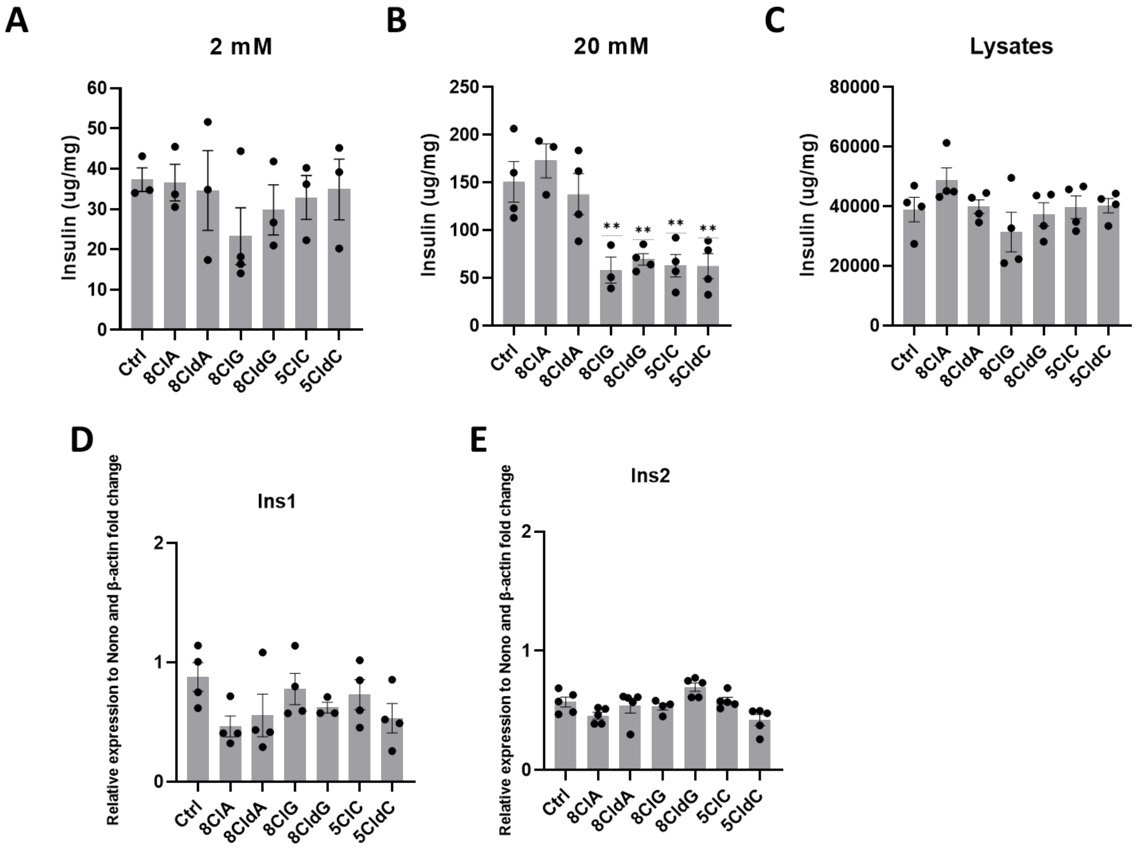

Figure S4. Effect of chlorinated nucleosides on insulin secretion in INS-1E cells. The INS-1 cells (1.2×10^5 cells/well) were exposed to 8ClA, 8CldA, 8ClG, 8CldG, 5ClC, and 5CldC (20 µM) for 24 h at 37 °C. Insulin secretion (A and B) was measured under basal low glucose conditions (2 mM) and under stimulation with high glucose conditions (20 mM) by ELISA. ELISA was also used to determine the insulin in cell lysates (C). In each case, results are normalized to protein concentration and represent the mean ± SEM of n = 3-4 experiments. The expression of Ins1 (D) and Ins2 (E) genes was assessed using qPCR. Results are expressed as the fold change compared to untreated control following normalization to housekeeping genes β-actin and Nono and represent the mean ± SEM of n = 4-5 experiments. Significance compared to untreated control was determined by one-way ANOVA with Dunnett's post hoc test, $*p<0.05$, $**p<0.01$.
